# Supplementary material for: “Let him die. He caused it”: A qualitative study on cancer stigma in Tanzania
Source: PLOS Glob Public Health. 2024 Jun 12;4(6):e0003283. doi: 10.1371/journal.pgph.0003283 (PMC11168623; doi:10.1371/journal.pgph.0003283)
Supplement: Attachment — Submitted filename: RESPONSE TO EDITOR AND REVIEWER COMMENTS_ 2024.docx [file pgph.0003283.s003.docx]

**EDITOR’S COMMENTS**

1. The scope of the issue is inconsistently addressed throughout the document.

*Thank you for your feedback. We have carefully reviewed and revised the relevant sections in the document to ensure consistency in addressing the scope of the issue. Our goal was to present the issue more cohesively.*

*To achieve this, we made some changes throughout the document. For instance, we aligned the last paragraph of the introduction (page 6, last paragraph) with the Method section (page 7, lines 12-18) and the introduction to the Results section (Page 12, lines 10 to 12). We also maintained consistency throughout the Discussion section.*

2. Logical flow of the manuscript is lacking, e.g., First stigma. And then its outcome, i.e., discrimination and its types.

*We appreciate your feedback regarding the flow of the content. To address your concerns, we have carefully reorganized the manuscript to ensure a more cohesive and structured presentation. Specifically, we have revisited the order of topics, prioritizing a logical progression from the description of stigma (beliefs, attitudes, and behavior) to its effects and outcomes (how stigma affects treatment, care/support seeking) and finally to solutions (strategies to reduce stigma).*

*In the results section, we have first presented a table with a summary of the themes, then tackled the beliefs surrounding cancer. This is then followed by behaviors and attitudes towards cancer and cancer patients. These two sections answer the first research question- ( 1. In Tanzania, how do people talk about or behave around cancer patients?) Next, we have tackled the Impact of stigma on patients. This answers the second research question.- (2. Does stigma influence the way people seek support or treatment related to cancer in Tanzania?). Lastly, we have looked at the Strategies to address stigma. This answers the third research question- (3. What are some ways we can help people with cancer to overcome or cope with stigma?).*

*We believe these adjustments have significantly improved the overall coherence of the manuscript.*

3. The type of qualitative research is not described.

*Our study employed an applied thematic analysis approach by identifying predetermined areas of exploration and inductively coding qualitative data within them as we reviewed the information. We have more detail about this in the materials and methods section on page 6 and further description in the procedures( page 9 and 10).*

4. The steps taken in the analysis were missed.

*Thank you for your feedback. We have improved the data analysis plan to make it more comprehensive and effective. You can find the details of these enhancements on page 11. Please note that some of the data analysis steps were included in the data quality assurance process, which is explained on pages 10.*

5. The role of the third, the consequent authors, and the one stated in the methods section contradicts. Furthermore, the role of authors is lacking.

*Clarifications have been made for the roles of each team member on page 9 (last line) and page 10 (line 8).*

6. Most of the study participants are at stage IV. Hence, how did you manage social desirability bias?

*Social desirability is a common concern in qualitative research. We addressed this potential challenge by using interviewers who were not otherwise affiliated with the cancer center and by reassuring participants that their responses would not be attached to them personally when shared outside of the research team. This created a sense of trust and likely facilitated the open sharing of personally sensitive information.*

7. Lacks logical flow, and especially the results section only presents the age and the type of cancer; why?

*The quotations also included the participant's gender. We did not attach other demographics to the quotations to protect the confidentiality of the participants. We have also reviewed the paper thoroughly to ensure a clear and logical flow to the content.*

8. Again, the results section took wider space than its fair share.

*We appreciate your observation and have revisited the results section to ensure it is presented concisely while maintaining the necessary detail and clarity. We have removed the minor subthemes(e.g., hopelessness and negative body image) and condensed others. We have made necessary adjustments to streamline the content, focusing on essential findings and adhering to the allocated space more appropriately.*

9. How do we compare a one-day experience with a 180-day experience ?

*Thank you for this important question. As noted in the Methods, we enrolled participants diagnosed with cancer at least 30 days prior but not more than 180 days before enrollment. We believe this window represents a time when the participant was likely still adjusting to a cancer diagnosis while allowing at least 30 days to absorb the emotional challenge of living with cancer.*

10. Maintain consistency, clarity and ensure whether the contents of all the sub sections contains all the contents

*We have taken the necessary steps to resolve this and can confirm that it has been successfully addressed.*

11. The data quality assurance methods are incomplete.

*Based on your feedback, we have reviewed the data quality assurance section (page 9&10) and added information to ensure completeness and thoroughness.*

*We have made some updates to our report. Specifically, we have incorporated our strategy for assessing the accuracy and saturation of our data in the data quality assurance section. Additionally, we have provided further details on how we enhanced the credibility of our results through peer rechecking, which can be found on page 10 in the last paragraph. Furthermore, we have highlighted the data triangulation method we used in our analysis ( Page 10, second paragraph). We used multiple researchers in the data analysis phase to strengthen the reliability and validity of our study findings.*

12. How can we extrapolate the Infectious disease-related stigma with the non-infectious one and try to revisit the framework meticulously?

*We greatly appreciate your comment. We have incorporated a new paragraph into our discussion section (page 25) which utilizes the HIV stigma framework developed by Earnshaw and Chadoir, and also cites our recently published work validating the stigma framework and assessment used in this study. Your suggestion has encouraged us to expand on the similarities and distinctions between infectious and non-infectious disease-related stigma. Furthermore, we have provided recommendations for future research and interventions.*
