# Supplementary material for: “Let him die. He caused it”: A qualitative study on cancer stigma in Tanzania
Source: PLOS Glob Public Health. 2024 Jun 12;4(6):e0003283. doi: 10.1371/journal.pgph.0003283 (PMC11168623; doi:10.1371/journal.pgph.0003283)
Supplement: Attachment — Submitted filename: Response to reviewers.docx [file pgph.0003283.s004.docx]

1. **The scope and the inner content of the article are inconsistent.**

*Thank you for your feedback. We've carefully reviewed the article and adjusted ensure consistency. We believe the current version reflects the intended scope and content.*

2. **Is the main objective of the study stigma or discrimination? If so, are you discussing the occult or overt character? ideal or practical?**

Thank you for your insightful comments and questions regarding our study on cancer-related stigma among individuals in Moshi, Tanzania. Our primary objective in this research is to explore cancer-related stigma within this specific context. We aim to delve into the beliefs, attitudes, and behaviors surrounding cancer and its impact on support-seeking behaviors among individuals affected by cancer in Moshi.

Our study encompasses both aspects of the distinction between stigma and discrimination. We explore various forms of stigma, including internalized, enacted, and anticipated stigma, as well as discrimination and social isolation experienced by individuals with cancer. Through qualitative analysis, we have identified prevalent beliefs contributing to cancer stigma, such as the misconception that cancer is a death sentence or contagious, as well as the belief in its costly treatment. Furthermore, we have examined behaviors and attitudes toward people with cancer, highlighting instances of social isolation, discrimination, and fear within the community. These findings underscore the complex interplay between societal perceptions and the experiences of individuals grappling with cancer in Moshi.

In terms of the occult versus overt character of stigma, our study primarily focuses on the overt manifestations of stigma, including social isolation, discrimination, and negative attitudes toward individuals with cancer. However, we acknowledge the nuanced nature of stigma, which may manifest subtly, such as internalized beliefs and self-stigmatization. Our analysis captures both explicit and implicit forms of stigma to comprehensively understand its impact on individuals' lives.

As for the ideal versus practical dimension, our study strives to bridge the gap between theoretical frameworks and real-world implications. While we draw upon established concepts of stigma, such as Weiss and Ramakrishna's definition, our ultimate goal is to inform practical interventions tailored to the unique challenges faced by individuals with cancer in low-resource settings like Moshi. By identifying specific beliefs contributing to stigma and exploring strategies to address them, we aim to inform the development of targeted psychosocial interventions that can effectively mitigate the burden of cancer-related stigma in this context.

To that end, we have written a paragraph in the introduction section to clarify this.

*“This study investigates cancer-related stigma and discrimination among individuals in Moshi, Tanzania. It identifies various forms of stigma experienced by individuals with cancer, including internalized, enacted, and anticipated stigma. We aim to highlight the complex interplay between societal perceptions and individual experiences of cancer-related stigma. The study aims to inform practical interventions that address the unique challenges faced by individuals with cancer in low-resource settings by identifying specific beliefs contributing to stigma and exploring strategies to address them. “*

Thank you once again for your valuable feedback, which has helped clarify the focus and scope of our study.

3. **The purpose of the article is stigma in the health care settings. However , it also includes individual, family and societal attitudes regarding the cancer patients among the populations. Hence I recommend you to make it one.**

It appears that there might be a slight misunderstanding regarding the focus of our study. We would like to clarify that our research aims to explore the broader spectrum of cancer-related stigma experienced by individuals in Moshi, Tanzania. While healthcare settings are indeed a component of this exploration, our study encompasses a comprehensive examination of stigma across various dimensions, including individual, family, and societal attitudes towards cancer patients within the community.

We believe that by investigating stigma in these diverse contexts, we can provide a more nuanced understanding of the challenges faced by individuals affected by cancer in low-resource settings. We aim to shed light on the complex interplay between individual beliefs, familial dynamics, and societal perceptions, thereby offering valuable insights into the multifaceted nature of cancer-related stigma.

4. **The comments in the methods section is not well addressed. E.g. Data quality assurance and lacks reference for example Guba 1972. Hence, the methods section still needs major revision.**

We have carefully addressed the comments, including bolstering the data assurance aspect. Upon thorough examination, we have ensured that all key components, such as study design, participant information, procedure, variables and measures, data analysis, ethical considerations, and limitations, are clearly outlined. However, we couldn't find a reference to Guba 1972 in our literature review or references section. If there are specific concerns or recommendations regarding this reference, please let us know so we can address them accordingly. We appreciate your feedback and are confident that the revisions have strengthened the rigor and clarity of our methods section.

5. **The philosophic stance of the researcher is out of the philosophy of qualitative study.
   -The type of qualitative study
   -The duration of the interview etc**

Our study adopts a qualitative approach, rooted in phenomenology, aiming to understand the lived experiences and perceptions of individuals facing cancer-related stigma in Moshi, Tanzania. The philosophical foundation of phenomenology aligns with our goal of exploring the subjective realities of participants, allowing for an in-depth examination of the complexities surrounding cancer stigma within the cultural and social context of Moshi.

Regarding the type of qualitative study, we conducted semi-structured interviews to gather rich narrative data from participants. These interviews were designed to elicit detailed accounts of participants' experiences, beliefs, and attitudes towards cancer stigma, offering valuable insights into the lived realities of individuals affected by cancer in the community.

As for the duration of interviews, each session typically lasted between 45 minutes to an hour, allowing sufficient time for participants to share their stories and perspectives in-depth while ensuring that the research process remained respectful of their time and comfort.

We value the reviewer's attention to methodological details and hope this clarification addresses their concerns about our study's philosophical stance and methodology.

*We have added a paragraph in the method section that clarifies this on page 6/7*

6. **The result, discussion, and consequent section need major and critical revision. They should also be short and precise.**

Thank you for your feedback. We have revisited the result, discussion, and consequent sections, making the necessary adjustments to ensure they effectively communicate the key points. We believe these revisions have significantly improved their clarity and conciseness.

7. **The reference needs a revisit.**

We have revisited the reference section to ensure accuracy and completeness. We appreciate your attention to detail and are committed to ensuring the quality of our references.

**Journal Requirements:**

1. **Please send a completed 'Competing Interests' statement, including any COIs declared by your co-authors. If you have no competing interests to declare, please state "The authors have declared that no competing interests exist". Otherwise please declare all competing interests beginning with the statement "I have read the journal's policy and the authors of this manuscript have the following competing interests:"**

We have now included a Competing Interests statement. Thank you.

**2. We have noticed that you have uploaded Supporting Information files, but you have not included a list of legends. Please add a full list of legends for your Supporting Information files after the references list.**

Thank you for bringing this to our attention. We have now included the list of legends for the Supporting Information files after the references list, as requested. Please let us know if there are any additional adjustments needed or if you have any further feedback.
