# Supplementary material for: “Let him die. He caused it”: A qualitative study on cancer stigma in Tanzania
Source: PLOS Glob Public Health. 2024 Jun 12;4(6):e0003283. doi: 10.1371/journal.pgph.0003283 (PMC11168623; doi:10.1371/journal.pgph.0003283)
Supplement: Attachment — Submitted filename: RESPONSE TO EDITOR AND REVIEWER COMMENTS[65].docx [file pgph.0003283.s002.docx]

**EDITOR’S COMMENTS**

1. Please include a complete copy of PLOS’ questionnaire on inclusivity in global research in your revised manuscript.

*Thank you. We have added the questionnaire on inclusivity to the supporting documents.*

2. We noticed that you used "unpublished" in the manuscript. We do not allow these references, as the PLOS data access policy requires that all data be either published with the manuscript or made available in a publicly accessible database. Please amend the supplementary material to include the referenced data or remove the references.

*We have replaced the referenced unpublished paper with its recently published version of the paper ( Reference# 18)*

3. In the online submission form, you indicated that "The de-identified data for this analysis may be made available upon request with an appropriate data transfer agreement". All PLOS journals now require all data underlying the findings described in their manuscript to be freely available to other researchers, 1. In a public repository, 2. Within the manuscript itself, or 3. Uploaded as supplementary information.
This policy applies to all data except where public deposition would breach compliance with the protocol approved by your research ethics board. If your data cannot be made publicly available for ethical or legal reasons (e.g., public availability would compromise patient privacy), please explain your reasons by return email and your exemption request will be escalated to the editor for approval. Your exemption request will be handled independently and will not hold up the peer review process but will need to be resolved should your manuscript be accepted for publication. One of the Editorial team will then be in touch if there are any issues.

*We are unable to make these data available for public deposition because open data sharing was not specified in participant informed consent, nor the approved ethical protocols for this study. We also have concerns about patient confidentiality in the sharing of detailed qualitative data, as the patient population at this cancer center is quite small. The de-identified data for this analysis may be made available upon reasonable request from the authors with an appropriate data transfer agreement.*

**REVIEWERS' NOTES AND COMMENTS**

**REVIEWER: 1**

The review is well written with obviously no mistakes. There is some mistyping in the references section: reference # 5 starts with (36.), which should be deleted; reference number 22 should specify the version used; and references on page 35, the font was changed.

*Thank you for this comment. We have now rectified the errors on references #5, #22, and the fonts on page 35.*

**REVIEWER #2:**

 The manuscript fails to meet:
1. The submission guideline of the journal

*We have taken your feedback into consideration and have successfully included the Inclusivity questionnaire while adhering to the provided guidelines. Thank you for your valuable input.*

2. Establishing a knowledge gap

*We sincerely appreciate your comment. One of the primary objectives of our study was to gain a more profound understanding of cancer stigma within the context of Moshi, which is a resource-limited setting. In many African settings, there is a rising incidence of cancer and unfortunately, poor outcomes, but the mental health aspects of cancer have often been overlooked and given lower priority. Furthermore, there is a noticeable dearth of research on cancer-related stigma with a specific focus on Africa.*

*Through our research, we aimed to shed light on how cancer stigma impacts the way patients in this region seek support from their communities and engage with treatment. Remarkably, there are currently no existing studies in this area for this region.*

*To provide further clarification on this topic, we have included additional information on page 5 of our study. Your feedback is greatly appreciated as it allows us to enhance the clarity and comprehensibility of our research. Thank you for your interest and engagement with our work.*

3. Appropriate study design and its details including the type of study design for instance in the abstract section.

*Thank you. We have added a statement in the abstract clarifying that we employed a cross-sectional qualitative research design.*

4. Data quality assurance

*We have included a new section (2.5) to address data quality assurance.*

*“To maintain high-quality data, all data was collected following clearly defined protocols. All data were collected by Bachelor-trained RAs with prior data collection experience who received initial training from the study’s co-principal investigator (BAK), a mental health provider with expertise in qualitative research and extensive experience working in Tanzania. The full study team met weekly to discuss study progress and to address any challenges to recruitment and data collection. Refresher training was provided to the RAs where necessary. All data were stored in REDCap with instituted access protocols to safeguard from unauthorized access or corruption. The team conducted weekly data audits to ensure that they met quality standards throughout the study period.”*

***Additionally, in the Data Analysis section, we explained how we maintained data quality during analysis.***

*“We randomly selected a subset of 28 sets of responses (20%) for the qualitative items to be re-coded by a second reviewer and evaluated for inter-coder agreement using a pre-established threshold of 80% agreement (24). Inter-coder agreement on these responses was 83.2%, which exceeded the desired threshold, and disagreements identified in the re-coding process were discussed and reconciled by the two reviewers until a consensus was reached.”*

*“During the coding process, we developed analytical code-based memos to identify patterns and salient themes across the data. Data matrices, reduction, and summary tables were used to synthesize the large number of files under study and to establish and identify any links among emerging themes. These would be discussed weekly and new observations recorded.*

6. Grammar, language, and statistics. 7. Inconsistent from the beginning to the end of the manuscript

*Thank you. We have thoroughly reviewed and proofread the manuscript to address this concern. Your feedback is greatly appreciated.*

**REVIEWER #3:**

Study design:
- Because the study focuses on stigma, I would want to know how participants were approached during recruitment and if selected for a specific subset of participants.

*Great questions. The study included participants who were all attendees at the Kilimanjaro Christian Medical Centre's cancer clinic. Our primary focus was to explore the lived experiences of these patients about their cancer and their journey in seeking care. Apart from adhering to specific eligibility criteria, which encompassed the timeframe since their diagnosis, their age, their capacity to provide consent both physically and cognitively, their ability to participate in the interview, and their proficiency in either Swahili or English, no additional specific selection criteria were applied and all eligible patients presenting to the clinic during the study period were invited to participate.*

*During their regular visits to the Cancer Care Centre, clinic nurses would initially identify individuals who met the eligibility criteria at the clinic's check-in area. Subsequently, they would provide these eligible individuals with basic information regarding the study. Those who expressed interest were then directed to a private office within the clinic, where they could meet with a research assistant to further discuss their participation in the study.*

*(See Pages 7&8)*

Was monetary motivation the principal factor for participants to join the research?

*The principal investigator has extensive experience working and conducting research in Tanzania, and the monetary incentive provided, which amounted to USD $2.25, is considered the standard compensation for participating in most brief research studies in this setting. This amount was approved by the local IRB and the National Institute for Medical Research and is considered enough to be a reasonable token of appreciation, but not excessive, nor likely to coerce uninterested patients to participate.*

Figures 1 and 2 are missing

*They have now been separately uploaded to the supplementary documents*.

Results:

"Th responses of the study participants" and several other spelling errors

*Thank you. They have now been corrected.*

In the "Beliefs Contributing to Cancer Stigma" in the "Death sentence, associated with
death" section, a different quote than the one above would make a stronger point, especially if this sentiment is found with 78% frequency. Repeating quotes is useful but can make a stronger point by alluding to the quote already presented and providing a different participant quote illustrating the same theme.

***Thank you for this observation. We have replaced the repeated quote with a new quote by a 37-year-old male prostate cancer patient:***

*“Having cancer is the end of life. When you pass by people who know you have cancer, they say that you will soon be dead.”*

"Negative body image and self-hate" the quote shared talks specifically about skin cancer. Does this theme extend beyond skin cancer? and if yes, how it is represented?

*Thank you for your keen observation and insightful input. In our study, we identified a minor theme related to negative body image and self-hate, which was expressed by only five out of the 140 participants. Notably, this theme extended beyond skin cancer to other forms of physical changes that can occur from cancer, and there was no discernible pattern as the cancer types associated with this theme varied. To enhance clarity, we have included an additional paragraph under the "Impact of Stigma" section on page 21.*

Did the research try to understand where these notions of stigma stem from? Ex: why do community members believe cancer to be contagious? This could be an interesting discussion to really get at different solutions (besides education) to advancing cancer care and diminishing stigma.

*This research primarily focused on the expression of cancer stigma, its impact on healthcare-seeking behavior, and potential strategies for stigma reduction. It did not delve into the origins of stigma. However, in the future, we aspire to investigate the root causes of stigma and conduct intervention studies aimed at reducing it.*

How do the author propose the emotional burden of care for patients can be reduced? Similarly, how can social support for cancer patients be promoted to increase access to care?

***Thank you for raising these questions. We have added two paragraphs on page 28 to address these two issues.***

*“The emotional burden of caregiving can be alleviated through the establishment of support groups for both caregivers and patients. These groups serve as safe spaces where individuals can openly share their fears, concerns, and anxieties, with the added benefit of finding practical solutions. Furthermore, resources like counseling provided by mental health professionals should be directed toward cancer patients and their caregivers, with an emphasis on making these services more accessible.”*

Addressing social support to improve access to care.

*“Enhancing social support for cancer patients can positively impact their treatment-seeking behavior and overall access to care. This can be achieved through various means, such as establishing peer support groups to provide patients with encouragement and companionship. Furthermore, raising community awareness about the significance of supporting cancer patients, whether through media campaigns or community outreach efforts, can help reduce stigma and foster a more supportive environment for these individuals***.”**
